# Standardized versus Individualized Acupuncture for Chronic Low Back Pain: A Randomized Controlled Trial

**DOI:** 10.1155/2013/125937

**Published:** 2013-10-28

**Authors:** Daniel Pach, Xiaoli Yang-Strobel, Rainer Lüdtke, Stephanie Roll, Katja Icke, Benno Brinkhaus, Claudia M. Witt

**Affiliations:** ^1^Institute for Social Medicine, Epidemiology and Health Economics, Charité—Universitätsmedizin Berlin, 10098 Berlin, Germany; ^2^Karl and Veronica Carstens Foundation, 45276 Essen, Germany; ^3^Center for Integrative Medicine, University of Maryland School of Medicine, Baltimore, MD 21201, USA

## Abstract

We aimed to compare the effectiveness of standardized and individualized acupuncture treatment in patients with chronic low back pain. A single-center randomized controlled single-blind trial was performed in a general medical practice in Germany run by a Chinese-born medical doctor trained in western and Chinese medicine. One hundred and fifty outpatients with chronic low back pain were randomly allocated to two groups (78 standardized and 72 individualized acupuncture). Patients received either standardized acupuncture or individualized acupuncture. Treatment encompassed between 10 and 15 treatments based on individual symptoms with two treatments per week. The main outcome measure was the area under the curve (AUC) summarizing eight weeks of daily rated pain severity measured with a visual analogue scale (0 mm = no pain, 100 mm = worst imaginable pain). No significant differences between groups were observed for the AUC (individualized acupuncture mean: 1768.7 (95% CI, 1460.4; 2077.1); standardized acupuncture 1482.9 (1177.2; 1788.7); group difference, 285.8 (−33.9; 605.5) *P* = 0.080). In this single-center trial, individualized acupuncture was not superior to standardized acupuncture for patients suffering from chronic pain. As a next step, a multicenter noninferiority study should be performed to investigate whether standardised acupuncture treatment for chronic low back pain might be applicable in a broader usual care setting. This trial is registered with ClinicalTrials.gov NCT00758017.

## 1. Introduction

In Western countries, chronic low back pain is a major health concern affecting the quality of life and productivity. Low back pain has a high economic impact. More than 70% of the population in industrialised countries is affected by low back pain [1]. In the United Kingdom, low back pain accounts for 13% of absences due to illness. The annual incidence in adults is up to 45%, with those aged 35–55 years affected most often. Although 90% of the episodes of acute low back pain settle within six weeks, up to 7% of patients develop chronic pain [1]. For chronic low back pain, a wide range of treatment options are available [2] although their efficacy is not always clear. A multimodal approach is recommended including providing information and counseling, exercise, pain therapy, behavioral therapy, and physiotherapy [2–4]. However, long-term effects are difficult to achieve [4]. 

Complementary and alternative medicine (CAM) therapies are widely used [5–10], and acupuncture was shown to be useful for chronic low back pain [11–14]. The acupuncture treatment costs are reimbursed by the German statutory health insurance companies [15]. However, the question remains whether individualized acupuncture, which needs more training and experience, is necessary to improve pain compared to a standardized acupuncture.

The practice of acupuncture has traditionally been based on the Chinese medical system of diagnosing “patterns of disharmony” where identifying the pattern determines the appropriate treatment principle [16]. Treatment principle, in turn, purportedly influences the treatment given, including the specific modalities used and acupoints stimulated. From the perspective of the Chinese medicine, patients with a single condition as defined by the western biomedicine may have one of several Chinese medical patterns, each of which requires a different treatment [17]. According to a study by Hogeboom et al., Chinese medical diagnoses and treatment recommendations for specific patients with chronic low back pain vary widely across practitioners [17]. They conclude that a comparison of individualized treatment with a thoughtfully developed standardized approach is warranted to determine which, if either, is superior [17]. A more standardized formulaic approach with a fixed set of points based on the best evidence might have the potential of improving the quality and efficiency of treatment and can support the integration of acupuncture into conventional care. At present, in China, standardization of acupuncture is strongly encouraged. For diagnoses such as stroke formulaic approaches are already well established [18].

The aim of our randomized controlled trial is to compare a standardized acupuncture that is based on evidence from previous acupuncture studies with individualized acupuncture based on the theory of Chinese medicine in patients with chronic low back pain.

## 2. Methods

### 2.1. Design

We performed a randomized controlled single-blind trial with treatment duration of eight weeks and a total observation time of 26 weeks per patient to compare the effectiveness of standardized with individualized acupuncture. Participants were blinded to group allocation.

This study followed the standards of the Declaration of Helsinki (revised version, Somerset West (SA), 1996 [19]) and the ICH-GCP guideline and was approved by the Ethics Committee Charité—Universitätsmedizin Berlin (Approval no. EA1/098/08). All patients gave oral and written informed consent.

### 2.2. Participants and Setting

Patients were recruited from the regular patients of a general medicine practice in Berlin, Germany, run by a Chinese-born medical doctor trained in western and Chinese medicine. The MD usually provides both conventional care and acupuncture to her patients. The acupuncture is usually individualized based on the Chinese medicine syndrome diagnosis. Patients with chronic low back pain suitable for acupuncture therapy (which is reimbursed by the German health insurances) were invited to participate in the study. No additional allowance was paid for the study. Participants were informed about the study using the following descriptions for both interventions: one group receives acupuncture according to individually selected points on the basis of diagnostics of Chinese medicine and the other group receives acupuncture consisting of acupuncture points that have shown their effectiveness in several studies.

The randomization sequence was generated by a data manager, who was not involved in the analysis of the data and enrolment of the patients, with Microsoft Office Excel 2003 in a 1 : 1 ratio stratified for gender. The list was integrated into a secured database (Microsoft Office Access 2003) and was not accessible to the other staff members or the study physician. Randomization took place in the practice using the secured database. The patient's allocation to the different treatment groups and the patient identification number for each single patient were assigned and accessible for the enrolling physician after patient data such as name and date of birth was entered and saved in the secured database. With that approach, the randomization list was hidden in the database and not accessible for anyone participating in the enrolment.

Patients were eligible for the trial if they fulfilled the following inclusion criteria: age of at least 18 years, male or female, low back pain for at least 3 months (clinical diagnosis of chronic low back pain confirmed by a medical specialist) and indication for treatment of low back pain with acupuncture confirmed by a medical specialist, average pain intensity of the last 7 days more or equal to 40 mm measured by a visual analogue scale (VAS 0–100 mm), intellectual and physical ability to participate in the study, and informed consent.

Main exclusion criteria were acupuncture during the last 6 months, start of a new therapy for low back pain within the last 4 weeks, pregnancy, substance or drug abuse, and participation in another clinical trial.

### 2.3. Intervention

All patients received Chinese medicine diagnostics including examination of pulse and tongue to avoid a bias due to a possible placebo effect caused by this kind of examination. Both acupuncture interventions were applied by the same medical doctor specialized in western general medicine (25 years of clinical practice) and trained in Chinese medicine with 20 years' experience in treating low back pain with acupuncture. According to the current statutory health insurance benefit catalogue, 10 to 15 treatment sessions per year are usually reimbursed. In our study, two treatment sessions per week had to be applied, with a maximum number of 10 to 15 sessions depending on the patient's individual needs. The standardized acupuncture was based on the acupuncture intervention from a large multicenter trial previously performed by our group [13, 20], developed by a large and systematic expert consensus [21]. From this trial's database, we determined the most frequently used points. Two Chinese medicine experts (BB and XYS) with more than 15 years of experience in acupuncture finalized the standardized treatment protocol used for the present study. Only body-needle acupuncture without electrical stimulation was allowed. Standardized acupuncture used the following points: (1) local points Bl 23, 24, and 25 and (2) distant points Bl 40, Bl 60, Gb 34, and K 3 in each session on both sides of the body. Individualized acupuncture was based on syndrome diagnosis, which was done before each treatment session. However, not more than 14 needles were applied to be comparable with the group with standardized acupuncture. For this study, we purchased Viva Sterile Acupuncture Needles, for single use only, pyrogen free, from Oxford Medical Supplies Ltd., Fairford, Gloucestershire, England. They had a needle length of 20 to 40 mm and a diameter of 0.2 to 0.3 mm. They were vertically inserted 1-2 cm deep into the skin depending on the size of the respective muscle. The needles were manually stimulated by rotation and lift-thrusting until a deqi sensation was reached. The needle retention time was about 25 min in both groups.

Because it was a trial in a real-life setting, comedication was allowed in both groups, and their intake was documented using diaries.

### 2.4. Outcome Measurements

The primary outcome measure was the area under the curve (AUC) summarizing the average low back pain intensity over eight weeks. For this, the back pain intensity of the last 24 hours was rated daily in a diary using a visual analogue scale [22] (VAS, 0–100 mm, 0 = no pain, 100 = worst imaginable pain) and then summed up over 56 days.

Secondary outcome measures included the VAS for pain during the previous 7 days at eight and 26 weeks and the following outcomes at eight and 26 weeks: back function (Hannover Functional Ability Questionnaire, HFAQ; in German, Funktionsfragebogen Hannover Rücken) [23], general health related quality of life (SF-36) [24], days absent from work, mean number of treatment sessions, mean duration of treatment, and days with physical therapy because of back pain. The patient diary (baseline to week 8) was also used to calculate the number of days with pain medication between weeks one and eight. In addition, we evaluated the safety of the interventions (recording of adverse events at each visit through the treatment physician) and blinding (patient guess of intervention group at 8 weeks). Except for safety data and data in the diary, outcome data was obtained by a study nurse, who was not blinded to the treatment arm.

To assess the patients' and doctor's expectation for improvement due to the treatment before randomization, patients and doctors had to document their expectation of the therapy on categorical scales: “recovery,” “distinct improvement,” “slight improvement,” and “no improvement” as well as their assessment of the presumed therapy's effectiveness: “very effective,” “effective,” “small effect,” and “no effect.”

### 2.5. Statistical Analysis

The study was designed to detect a clinically relevant effect (standard mean difference of 0.5) for the primary outcome measure with a power of 80% and a significance level of 5% using a two-sided *t*-test. Based on that calculation, a total of 128 participants were needed. Taking about 20% potential drop-outs into account, 150 participants (75 per group) were planned to be included into the study. The primary analysis population was the intention to treat (ITT) population, based on the available data. Each randomized participant was included into the analysis regardless of the adherence to the assigned treatment. 

The primary outcome (daily low back pain intensity summed over 8 weeks) was evaluated using analysis of covariance (ANCOVA) including treatment group, with baseline value and participants' initial expectation from treatment as covariates. This resulted in adjusted mean severity scores per treatment group, 95% confidence intervals and *P* value for treatment group comparison. Secondary outcome parameters were analysed by similar ANCOVA or generalized estimating equation (GEE) models in a similar fashion. Missing data were not imputed. All tests were two-sided; the significance level for the primary outcome was set at 0.05, and all other *P* values were considered explorative. Analyses were performed in SAS Version 9.1 (SAS Institute, Cary, NC, USA). 

## 3. Results

### 3.1. Participants and Treatment

**Figure 1:**
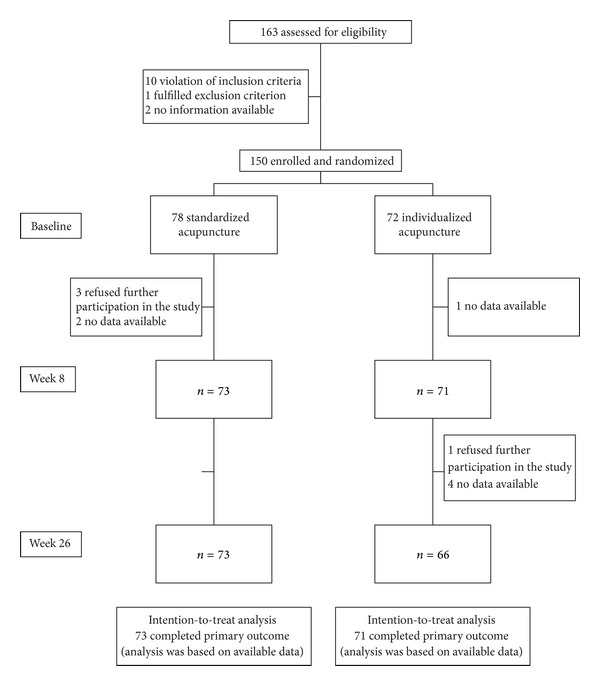
Recruitment, treatment, and follow-up of patients with chronic low back pain.

From 163 possible participants screened, 150 were enrolled between January 2009 and January 2011 (Figure 1) and randomized into the two groups (standardized group *n* = 78, individualized group *n* = 72). The mean age was 57.8 ± 12.5 (mean ± sd) years, 58% were female and the mean duration of symptoms was 16.3 ± 12.3 years. At baseline, the average pain intensity on the VAS was 58.5 ± 11.3 mm (for other baseline characteristics see Table 1).

The mean number of treatments was 10.4 ± 2.8 in the standardized group and 11.0 ± 2.5 in the individualized group (median 10.0 and 10.0, resp.). Six patients were lost to follow-up at week eight but were included in the ITT analysis. Follow-up data after 26 weeks was available for 139 patients (standardized group *n* = 73, individualized group *n* = 66). The reasons for missing follow-up data are shown in Figure 1.

### 3.2. Outcomes

**Figure 2:**
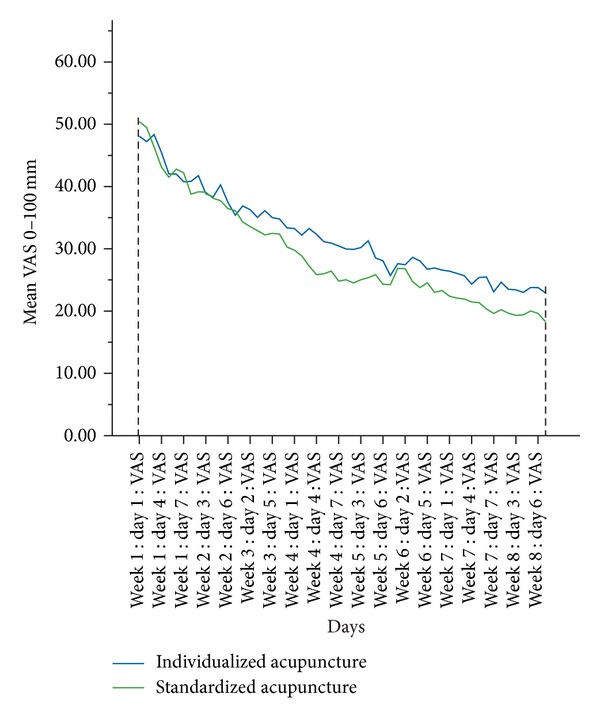
Mean symptom severity VAS of daily data over 8 weeks, nonadjusted data. Dashed lines represent the borders of the area under the curve.

Both groups showed a clinically meaningful improvement [25] after 8 weeks regarding pain severity (Figure 2). The primary endpoint, the area under the curve (AUC) for the pain severity from baseline to end of week 8, was comparable between both groups (Table 2, Figure 2 for unadjusted data) and showed no statistically significant differences (adjusted group difference, 285.8 (95% CI −33.9; 605.5); *P* = 0.080, Table 2). 

**Figure 3:**
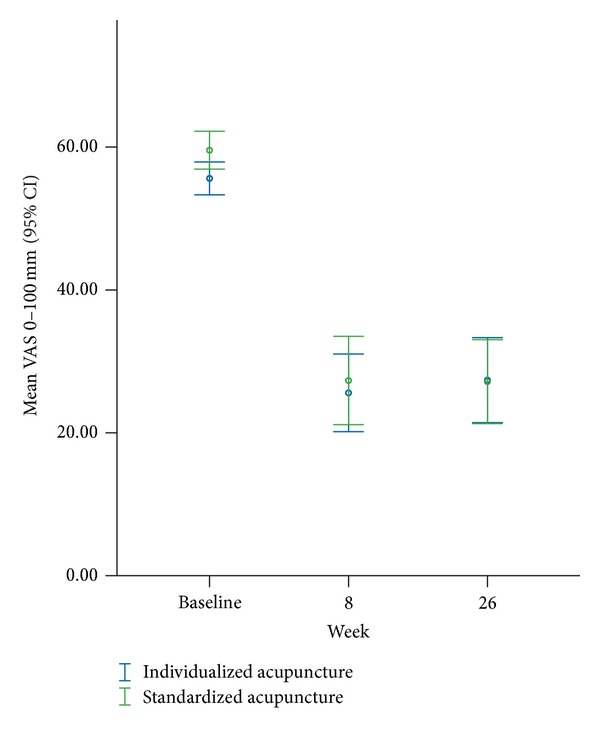
Mean (with 95% confidence interval) pain intensity over the last 7 days (VAS) at week 8 and at week 26, nonadjusted data. At baseline, VAS was different between both groups (0.014), but not for the latter time points.

Secondary outcomes showed consistent results. The average pain severity after 8 weeks and 26 weeks did not differ significantly between both groups (Table 2, Figure 3 for unadjusted data). Accompanying therapy including concurrent therapies was not significantly different between both groups regarding days with medication intake (week 1 to end of week 8), days with physical therapy because of back pain (week 1 to end of week 8), and number of therapy sessions and duration of therapy (baseline to end of therapy). Furthermore, for the secondary outcomes HFAQ, QoL, and sick leave days at week 8 and week 26, no significant group differences were observed (Table 2). 

Of the 150 patients in both intervention groups, none reported acupuncture-related side effects. However, adverse events reported by the patients included breast cancer, herpes zoster, and common cold (individualized group: 7 events, standardized group: 8 events), but none had a causal relation to the acupuncture treatment.

After the end of treatment, patients were asked to guess what treatment intervention had been administered to them. In the standardized group, 78.1% guessed they were in the standardized group while, in the individualized group, 55.7% guessed they were in the individualized group (Table 3). 

## 4. Discussion

In our study, we could not show a statistically significant difference between standardized and individualized acupuncture in the treatment of chronic low back pain. Results were consistent over all outcomes.

The main strengths of this trial are the randomized single-blinded study design, the relatively large sample size for a single-center trial on CAM, and the high compliance and follow-up rates. We aimed to answer a research question that has relevance for usual care practice. Therefore, the chosen setting in a general medical practice reflects a real-world setting. This routine care setting is a reason for the broad inclusion and exclusion criteria in our trial and the decision to leave the decision on the number of visits to the physician. The physician who performed the acupuncture usually treats her patients with individualized acupuncture. However, to evaluate the quality of care in her practice, she was highly motivated to compare it with a standardized acupuncture approach, which was comprised of those acupuncture points that were most frequently used by the participating physicians in a large randomized multicenter trial of acupuncture in patients with low back pain [20]. We think the fact that this was a single-center trial carried out by a single practitioner is both strength (reducing implementation variability) and a weakness (limiting generalizability).

The outcome measure VAS is a validated and sensitive tool, which is widely used to measure pain. By using the area under the curve summarizing the VAS of week 1 to week 8 as our primary outcome, we were able to include different time points into one primary outcome measure. However, this might have caused an underestimation of the treatment effect, because the measure averages the pain intensity of the whole treatment course. Using only week 5 to week 8 data would have been another option; however, secondary outcomes such as the pain measured after eight weeks and after 26 weeks showed also no significant differences between groups. The secondary outcome measures we included in our study such as medication intake, back function, and quality of life also confirmed the results. 

We tested sustained blinding in both treatment groups. The standardized group guessed the right treatment more often than one could expect by chance. One reason might be that more than half of the study patients were experienced with acupuncture. We do not think that this affected our results because both groups were informed to get an effective treatment and improved similarly. Furthermore, assessing blinding is a controversial discussion and was deleted from the current version of the CONSORT checklist [26].

The aim of this study was not to assess the efficacy of acupuncture for chronic low back pain. Berman et al. discussed its clinical relevance, [11] and a very recent patient-level data meta-analysis came to the conclusion that acupuncture is statistically significant superior to sham-acupuncture for chronic low back pain, [14] although the effect between groups was of small size. 

Another option for a research question would have been noninferiority trial to evaluate whether standardized acupuncture for chronic low back pain is noninferior to individualized acupuncture. However, we decided to follow a superiority hypothesis, because individualized acupuncture requires more time resources, both from a training and application perspective. A multicenter trial would have produced more generalizable results and reduced possible bias of the participating physicians regarding their favored therapy. 

Our study results suggest that there is no relevant difference in the outcome of standardized and individualized acupuncture in the treatment of chronic low back pain. For week 1 to week 8, one could even observe a trend toward superiority of the standardized acupuncture. However, this lack of statistical significance has to be interpreted with caution. Because of our statistical superiority approach, our study does not prove that standardized acupuncture is non inferior or equivalent to individualized acupuncture.

Our study might be compared with a study conducted in the United States that directly compared individualized acupuncture with standardized acupuncture [27]. They showed that performing a Chinese medicine diagnosis did not change the result that patients in all acupuncture groups (individualized, standardized, or sham) improved significantly more than patients receiving usual medical care, but the acupuncture groups did not differ significantly from one another [11]. Standardized acupuncture points in Cherkin's study were based on an expert consensus and comprised of eight points (individualized 11 points). Our standardized acupuncture was based on data from a large trial on low back pain and comprised 14 points. 

In conclusion, in this single-center trial, individualized acupuncture was not superior to standardized acupuncture for patients suffering from chronic low back pain. If a fixed set of points can be well established for a specific condition, this might have wide implications. Without the necessity for a diagnosis according to Chinese medicine, it might reduce time and knowledge necessary for the treatment. These can extend the availability of acupuncture toward conventional care. A next step multicenter noninferiority study to investigate whether standardized acupuncture treatment for chronic low back pain might be applicable in a broader usual care setting and is more cost-effective could have clinical and health policy implications. 

## Figures and Tables

**Table 1:** Baseline demographic and clinical characteristics of trial groups.

Characteristics	Standardized acupuncture (*n* = 78)	Individualized acupuncture (*n* = 72)
Age (years; mean ± sd)	59.3 ± 12.0	56.1 ± 12.9
Gender (*n* (%))		
Female	42 (53.8)	45 (62.5)
Male	36 (46.2)	27 (37.5)
BMI (kg/m²; mean ± sd)	27.2 ± 4.6	27.0 ± 5.0
>10 years of school (*n* (%))	10 (12.8)	24 (33.3)
Size of household (*n* (%))		
Single person	22 (28.2)	16 (22.2)
Multiperson	55 (70.5)	56 (77.7)
Average low back pain during the previous 7 days (VAS^§^; mean ± sd)	60.7 ± 12.0	56.2 ± 10.0
Duration of low back pain (years; mean ± sd)	16.8 ± 12.8	14.9 ± 11.8
Concomitant diseases (*n* (%))		
Diseases of the nervous system	0 (0)	2 (2.7)
Endocrine, nutritional, and metabolic diseases	1 (1.2)	3 (4.1)
Diseases of musculoskeletal system	12 (15.3)	4 (5.5)
Sick leave days of previous 8 weeks (days; mean ± sd)	8.9 ± 14.8	8.2 ± 13.5
Prior consultation because of low back pain (*n* (%))	78 (100)	69 (95.8)
Low back pain/disability (HFAQ^#^; mean ± sd)	36.0 ± 19.1	37.4 ± 20.4
SF-36 quality of life (SF-36^#^; mean ± sd)		
Physical health	34.7 ± 7.7	35.7 ± 9.3
Mental health	49.7 ± 11.1	46.2 ± 12.5
Experiences with acupuncture (*n* (%))	60 (76.9)	56 (77.7)
Expected effectiveness of acupuncture (*n* (%))		
Very effective	32 (41.0)	24 (33.3)
Effective	41 (52.5)	48 (66.6)
Less effective	4 (5.1)	0 (0)
Ineffective	0 (0)	0 (0)
Preference (*n* (%))^$^		
Standardized acupuncture	30 (38.4)	35 (48.6)
Individualized acupuncture	46 (58.9)	37 (51.3)

BMI: body mass index; VAS: visual analogue scale for assessing the average low back pain intensity; HFAQ: Hannover Functional Ability Questionnaire; SF-36: 36-item quality-of-life questionnaire.

^§^Lower values indicate better status.

^#^Higher values indicate better status.

^$^Missing answers add to 100%.

**Table 2:** Primary and secondary outcomes at 8 and 26 weeks (adjusted for baseline value and participant's expectation)*.

	Standardized acupuncture mean (95% CI)	Individualized acupuncture mean (95% CI)	Differences individualized versus standardized acupuncture (95% CI)	*P* value
Overall low back pain—area under the curve** (sum of daily VAS^§^): week 1 to 8^*µ*^	1,482.9 (1,177.2; 1,788.7)	1,768.7 (1,460.4; 2,077.1)	285.8 (−33.9; 605.5)	0.080
Mean overall low back pain: (mean daily VAS^§^): weeks 1 to 8^*µ*^	26.5 (21.0; 31.9)	31.6 (26.1; 37.1)	5.1 (−0.6; 10.8)	0.080
Days with pain medication: weeks 1 to 8^*µ*^	4.9 (0.4; 9.3)	5.6 (1.2; 10.0)	0.7 (−3.9; 5.4)	0.752
Days with physiotherapy: weeks 1 to 8^*µ*^	2.1 (0.1; 4.0)	1.9 (0.01; 3.8)	−0.2 (−2.2; 1.8)	0.867
Number of acupuncture therapy sessions	9.8 (8.4; 11.2)	10.3 (8.9; 11.7)	0.5 (−0.3; 1.3)	0.226
Duration of therapy (minutes per week)	41.1 (30.7; 51.5)	44.4 (34.4; 54.4)	3.3 (−2.6; 9.3)	0.272
Average low back pain during the previous 7 days (VAS^§^)				
8 weeks	27.4 (21.2; 33.7)	28.7 (23.4; 34.0)	1.3 (−5.8; 8.4)	0.723
26 weeks	27.3 (21.0; 33.7)	30.5 (24.6; 36.3)	3.1 (−4.5; 10.8)	0.424
Low back pain/disability (HFAQ^#^)				
8 weeks	25.8 (21.9; 29.6)	27.5 (22.8; 32.1)	1.7 (−3.4; 6.8)	0.513
26 weeks	24.3 (20.4; 28.3)	25.9 (21.0; 30.8)	1.5 (−3.7; 6.8)	0.569
SF-36 quality of life (SF-36^#^)				
Physical health at 8 weeks	42.7 (40.3; 45.1)	42.1 (40.1; 44.1)	−0.5 (−3.5; 2.4)	0.714
Physical health at 26 weeks	43.1 (40.7; 45.5)	41.7 (39.5; 43.8)	−1.5 (−4.5; 1.6)	0.343
Mental health at 8 weeks	49.5 (47.0; 52.1)	50.0 (47.4; 52.6)	0.4 (−2.7; 3.6)	0.788
Mental health at 26 weeks	48.8 (46.1; 51.6)	50.7 (47.9; 53.5)	1.9 (−1.6; 5.4)	0.287
Sick leave days				
8 weeks	4.8 (1.8; 7.8)	4.5 (1.5; 7.4)	−0.3 (−3.4; 2.8)	0.843
26 weeks (previous 4 months)	9.0 (3.6; 14.4)	9.7 (4.1; 15.2)	0.6 (−4.8; 6.0)	0.817

VAS: visual analogue scale for assessing the average low back pain intensity; HFAQ: Hannover Functional Ability Questionnaire; SF-36: 36-item quality-of-life questionnaire.

*The area under the curve was evaluated using analysis of covariance (ANCOVA) including treatment group, with baseline value and participants' initial expectation from treatment as covariates. Secondary outcome parameters were analysed by similar ANCOVA or generalized estimating equation (GEE) models in a similar fashion.

^*µ*^Based on daily data from a diary.

^§^Lower values indicate better status.

^#^Higher values indicate better status.

**The area under the curve (AUC) represents the sum of daily VAS scores (0–100) over 8 weeks.

**Table 3:** Guesses of group allocation.

Patients' guesses	Group assignment	
	Standardized	Individualized
Standardized	57 (78.1%)	39 (55.7%)
Individualized	16 (21.9%)	31 (44.3%)

*Chi-square test.
